# Sex Differences in the Incidence and Risk Factors of Myocardial Injury in COVID-19 Patients: A Retrospective Cohort Study

**DOI:** 10.3389/fphys.2021.632123

**Published:** 2021-02-16

**Authors:** Ran Cheng, Chuan Liu, Jie Yang, Yuanqi Yang, Renzheng Chen, Xiaohan Ding, Xubin Gao, Jingbin Ke, Fangzhengyuan Yuan, Chunyan He, Yang Shen, Limin Zhang, Ping Li, Hu Tan, Lan Huang

**Affiliations:** ^1^The Second Affiliated Hospital, Institute of Cardiovascular Diseases of People’s Liberation Army of China (PLA), Army Medical University, Chongqing, China; ^2^Department of Health Care and Geriatrics, People’s Liberation Army Joint Logistic Support Force 940th Hospital, Lanzhou, China; ^3^Department of Infectious Diseases, Huoshenshan Hospital, Wuhan, China

**Keywords:** COVID-19, sex differences, myocardial injury, risk-factors, inflammation, coagulation disorder

## Abstract

Male novel coronavirus disease (COVID-19) patients tend to have poorer clinical outcomes than female patients, while the myocardial injury is strongly associated with COVID-19-related adverse events. Owing to a lack of corresponding data, we aimed to investigate the sex differences in the incidence of myocardial injury in COVID-19 patients and to identify the potential underlying mechanisms, which may partly account for the sex bias in the incidence of adverse events. This retrospective study included 1,157 COVID-19 patients who were hospitalized in Huoshenshan Hospital from 12 March 2020 to 11 April 2020. Data on the patients’ demographic characteristics, initial symptoms, comorbidities and laboratory tests were collected. Totally, 571 (49.4%) female and 586 (50.6%) male COVID-19 patients were enrolled. The incidence of myocardial injury was higher among men than women (9.2 vs. 4.9%, *p* = 0.004). In the logistic regression analysis, age, and chronic kidney disease were associated with myocardial injury in both sexes. However, hypertension [odds ratio (OR) = 2.25, 95% confidence interval (CI) 1.20–4.22], coronary artery disease (*OR* = 2.46, 95% CI 1.14–5.34), leucocyte counts (*OR* = 3.13, 95% CI 1.24–7.86), hs-CRP (*OR* = 4.45, 95% CI 1.33–14.83), and D-dimer [*OR* = 3.93 (1.27–12.19), 95% CI 1.27–12.19] were independent risk factors only in the men. The correlations of hs-CRP and D-dimer with hs-cTnI and BNP were stronger in the men. The incidence of myocardial injury in COVID-19 patients is sex-dependent, predominantly in association with a greater degree of inflammation and coagulation disorders in men. Our findings can be used to improve the quality of clinical management in such settings.

## Introduction

As of November 2020, the novel 2019 coronavirus disease (COVID-19) has led to more than 55 million confirmed cases worldwide, including nearly 1.5 million deaths (World Health Organization, 2020b). The mortality associated with the disease ranges from 5.8 to 11.7% (Du et al., 2020; Mehra et al., 2020; Shi et al., 2020b). Studies focusing on the epidemiological and clinical characteristics of COVID-19 have shown that, in addition to old age and comorbidities, sex differences are also associated with disease deterioration and mortality (Chen et al., 2020; Yang et al., 2020), with male patients showing significantly higher mortality values (Epidemiology Working Group for NCIP Epidemic Response, 2020). In Spain, the mortality among male COVID-19 patients is twice as high as that among their female counterparts (Pastor-Barriuso et al., 2020). Another observational, longitudinal study on 10-year mortality, enrolled 1,284 subjects without COVID-19, demonstrated that males tended to have a lower prevalence of frailty and comorbidities, receive fewer drugs, but have higher mortality than females (Corbi et al., 2019). However, there remains a lack of clarity on the underlying reasons for the sex differences in the incidence of fatal outcomes in such settings. In COVID-19, mortality is strongly associated with the incidence of myocardial injury (7.2 to –27.8%) (Guo et al., 2020; Huang et al., 2020; Wang et al., 2020). Moreover, myocardial injury development may result in the deterioration of other COVID-19-related outcomes [e.g., acute respiratory distress syndrome (ARDS), intensive care unit (ICU) admission and ventilator therapy] (Lala et al., 2020; Lombardi et al., 2020; Shi et al., 2020b). Therefore, the sex differences in the incidence of COVID-19-related myocardial injury may partly account for the sex bias in the incidence of adverse events. While some studies showed that the incidence of myocardial injury is higher among men than women (Guo et al., 2020), others did not observe significant sex-related differences (Shi et al., 2020b). Thus, whether the incidence of COVID-19-related myocardial injury is sex-dependent remains controversial.

Therefore, we aimed to retrospectively compare the epidemiological characteristics, laboratory test results, and risk factors associated with myocardial injury between female and male COVID-19 patients to identify sex differences in the incidence of myocardial injury as well as the underlying potential mechanisms so as to facilitate optimal clinical management.

## Materials and Methods

### Study Design and Participants

A total of 1,201 patients who were hospitalized at Huoshenshan Hospital (Wuhan, China) from 12 March 2020 to 11 April 2020 and diagnosed with laboratory-confirmed COVID-19 according to World Health Organization guidelines (World Health Organization, 2020a) were enrolled in this single-center, retrospective cohort study. Patients (1) aged under 18 years, (2) without laboratory test data, or (3) without high-sensitivity cardiac troponin I (hs-cTnI) test results were excluded (Shi et al., 2020b). Real-time reverse transcriptase-polymerase chain reaction performed using throat swab specimen was employed for severe acute respiratory syndrome coronavirus 2 (SARS-CoV-2) infection detection.

Our study protocol was approved by the Human Ethics Committee, Huoshenshan Hospital (No. HSSLL023). The study conformed to the ethical guidelines of the Declaration of Helsinki. Given the limited medical resources and the need to treat a large volume of patients in the urgently constructed hospital in a short time, it was a huge challenge to gather the informed consent form for every hospitalized patient. Oral informed consent was approved by the ethics commission of the hospital for patients with COVID-19 (Shi et al., 2020a).

### Data Collection

Data on the patients’ demographics, initial symptoms, comorbidities, and laboratory tests (routine blood test, renal and liver function, coagulation profile, cardiac biomarkers, inflammatory biomarkers) were obtained from standardized clinical electronic medical records. Laboratory tests were completed within 1 day after admission. All data were independently verified and entered into the computer database by two experienced physicians.

### Definition

Age was classified as ≤65 years and >65 years (Du et al., 2020). Initial symptoms were defined as the first symptoms that appeared in the early infection stages. Comorbidities were diagnosed using the International Classification of Disease 10 codes before SARS-CoV-2 infection. Laboratory tests were classified as normal or abnormal based on Huoshenshan Hospital criteria (Table 2). ARDS was defined according to the Berlin Definition (Ranieri et al., 2012). Myocardial injury was confirmed if the hs-cTnI level was higher than the 99th percentile upper reference limit (Thygesen et al., 2018). According to the guidelines for diagnosis and management of COVID-19 (5th version, in Chinese) released by the National Health Commission of China, the severe and critically ill cases was defined when meeting any of the follows: respiratory rate ≥30 times/min; pulse oxygen saturation ≤93% at rest; arterial oxygen partial pressure/fraction of inspired oxygen ≤300 mmHg; respiratory failure requiring mechanical ventilation; or respiratory failure combined with other organ failure requiring ICU treatment (National Health Commission of China, 2020).

**TABLE 1:** Comparison of demographics, initial symptoms, and comorbidities between myocardial injury and without-myocardial injury in female and male COVID-19 patients.

	**Female**			**Male**		
	**Myocardial injury (*n* = 28)**	**Without-myocardial injury (*n* = 543)**	***p*-value**	**Myocardial injury (*n* = 54)**	**Without-myocardial injury (*n* = 532)**	***p*-value**
**Demographics**						
Age, years	67.5 (57.5–75.75)	61 (53–68)	0.004	72 (63.75–78)	61 (51–69)	<0.001
Current smoker, n (%)	0 (0)	1 (0.2)	1.000	5 (9.3)	67 (12.6)^##^	0.477
**Initial symptoms**						
Fever (≧37.3°C), n (%)	18 (64.3)	367 (67.6)	0.716	29 (53.7)	391 (73.5)^#^	0.002
Cough, n (%)	18 (64.3)	382 (70.3)	0.494	32 (59.3)	337 (63.3)^#^	0.553
Sputum, n (%)	2 (7.1)	67 (12.3)	0.599	10 (18.5)	69 (13.0)	0.255
Short of breath, n (%)	14 (50.0)	241 (44.4)	0.560	26 (48.1)	239 (44.9)	0.650
Fatigue, n (%)	11 (39.3)	196 (36.1)	0.732	13 (24.1)	187 (35.2)	0.102
Nausea/vomiting, n (%)	1 (3.6)	18 (3.3)	1.000	4 (7.4)	10 (1.9)	0.039
Stuffy/runny noses, n (%)	0 (0)	4 (0.7)	1.000	0 (0)	2 (0.4)	1.000
Throat discomfort, n (%)	0 (0)	27 (5.0)	0.452	1 (1.9)	10 (1.9)^##^	1.000
**Comorbidities**						
Hypertension, n (%)	15 (53.6)	193 (35.5)	0.053	35 (64.8)	177 (33.3)	< 0.001
Diabetes, n (%)	7 (25.0)	81 (14.9)	0.241	13 (24.1)	91 (17.1)	0.202
Arrhythmia, n (%)	1 (3.6)	24 (4.4)	1.000	4 (7.4)	20 (3.8)	0.353
Malignant neoplasms, n (%)	1 (3.6)	12 (2.2)	0.484	3 (5.6)	14 (2.6)	0.427
CAD, n (%)	2 (7.1)	37 (6.8)	1.000	14 (25.9)*	34 (6.4)	<0.001
COPD, n (%)	1 (3.6)	15 (2.8)	0.560	4 (7.5)	29 (5.5)^#^	0.758
CLD, n (%)	1 (3.6)	9 (1.7)	0.398	3 (5.6)	21 (3.9)^#^	0.835
CKD, n (%)	3 (10.7)	9 (1.7)	0.017	5 (9.3)	9 (1.7)	0.003
Anemia, n (%)	1 (3.6)	10 (1.8)	0.428	3 (5.6)	8 (1.5)	0.118
Cerebrovascular disease, n (%)	1 (3.6)	18 (3.3)	1.000	8 (14.8)	24 (4.5)	0.004
**Clinical outcomes**						
Respiratory failure, n (%)	10 (35.7)	20 (3.7)	<0.001	28 (51.9)	26 (4.9)	<0.001
ARDS, n (%)	11 (39.3)	15 (2.8)	<0.001	25 (46.3)	23 (4.3)	<0.001
ICU admission, n (%)	8 (28.6)	18 (3.3)	<0.001	25 (46.3)	25 (4.7)	<0.001
Death, n (%)	8 (28.6)	6 (1.1)	<0.001	19 (35.2)	9 (1.7)	<0.001
**Disease severity**						
Mild, n (%)	10 (35.7)	398 (73.3)	<0.001	20 (37.0)	362 (68.0)	<0.001
Severe and critically ill, n (%)	18 (64.3)	145 (26.7)		34 (63.0)	170 (32.0)	

Continuous variables with non-normal distribution were represented as medians (25th–75th percentile) and categorical data were represented as count and percentage. COVID-19, novel 2019 coronavirus disease; COPD, chronic obstructive pulmonary disease; CAD, coronary artery disease; CLD, chronic liver disease; CKD, chronic kidney disease; ARDS, acute respiratory distress syndrome; ICU, intensive care unit admission. *p < 0.05, compared to female patients with myocardial injury. ^#^p < 0.05, ^##^p < 0.01, compared to female patients without myocardial injury.

**TABLE 2:** Association between laboratory findings and myocardial injury in female and male COVID-19 patients.

**Variables**		**Female**				**Male**			
		**Univariable OR (95%CI)**	***p*-value**	**Multivariable OR (95%CI)**	*p*-value*	**Univariable OR (95%CI)**	***p*-value**	**Multivariable OR (95%CI)**	*p*-value*
Leucocytes, ×10^9^/L	4–10	1 (Ref)				1 (Ref)			
	<4 or >10	3.56 (1.61–7.86)	0.002	1.97 (0.59–6.58)	0.271	2.70 (1.48–4.94)	0.001	3.13 (1.24–7.86)	0.016
Neutrophil percentage, %	40–75	1(Ref)				1 (Ref)			
	<40 or >75	6.36 (2.80–14.41)	<0.001	2.90 (0.83–10.14)	0.095	9.07 (4.98–16.51)	<0.001	1.86 (0.71–4.85)	0.205
Hemoglobin, g/L	≥115	1 (Ref)				1 (Ref)			
	<115	1.87 (0.87–4.00)	0.109	0.34 (0.11–1.10)	0.073	3.47 (1.95–6.16)	<0.001	0.94 (0.38–2.35)	0.900
Platelets, ×10^9^/L	100–300	1 (Ref)				1 (Ref)			
	<100 or >300	1.81 (0.80–4.11)	0.156	0.83 (0.26–2.69)	0.761	2.65 (1.47–4.77)	0.001	1.77 (0.67–4.65)	0.247
hs–CRP, mg/L	≤4	1 (Ref)				1 (Ref)			
	>4	5.53 (2.39–12.82)	<0.001	2.21 (0.67–7.32)	0.193	16.40 (6.42–41.92)	<0.001	4.45 (1.33–14.83)	0.015
ALT, IU/L	≤50	1 (Ref)				1 (Ref)			
	>50	0.78 (0.18–3.4)	0.745	1.02 (0.17–5.96)	0.985	1.30 (0.67–2.51)	0.437	1.07 (0.40–2.87)	0.900
TBil, μmol/L	≤26	1 (Ref)				1 (Ref)			
	>26	5.30 (1.07–26.27)	0.041	7.38 (0.98–55.76)	0.053	3.84 (0.99–14.92)	0.052	3.88 (0.37–40.51)	0.257
BUN, mmol/L	≤9.5	1 (Ref)				1 (Ref)			
	>9.5	16.46 (4.85–55.82)	<0.001	4.81 (0.77–29.85)	0.092	21.76 (10.28–46.09)	<0.001	2.97 (0.85–10.43)	0.089
Creatinine, μmol/L	≤100	1 (Ref)				1 (Ref)			
	>100	1.90 (0.90–3.99)	0.092	0.93 (0.24–3.54)	0.914	6.11 (3.14–11.87)	<0.001	1.34 (0.34–5.24)	0.678
PT, s	≤18	1 (Ref)				1 (Ref)			
	>18	10.59 (1.84–60.81)	0.008	1.16 (0.10–13.29)	0.908	17.00 (5.77–50.06)	<0.001	5.58 (0.84–37.06)	0.075
APTT, s	≤40	1 (Ref)				1 (Ref)			
	>40	7.04 (1.35–36.82)	0.021	6.33 (0.47–85.51)	0.165	6.44 (2.02–20.50)	0.002	0.21 (0.02–2.01)	0.174
D-dimer, mg/L	≤0.6	1 (Ref)				1 (Ref)			
	>0.6	5.50 (2.14–14.12)	<0.001	2.78 (0.86–9.02)	0.089	12.87 (4.99–33.17)	<0.001	3.93 (1.27–12.19)	0.018

*OR, odds ratio; 95%CI, 95% confidence intervals. ^∗^Multivariable models were adjusted by age, history of hypertension, coronary artery disease, chronic kidney disease. hs-CRP, high-sensitive C-reactive protein; ALT, alanine aminotransferase; AST, aspartate aminotransferase; TBil, total bilirubin; hs-cTnI, high-sensitive cardiac troponin I; BNP, brain natriuretic peptide; PT, prothrombin time; APTT, activated partial thromboplastin time.*

### Statistical Analysis

Continuous variables were represented as medians (25th–75th percentile). Independent sample *t*-tests or Mann-Whitney *U* tests were used for the comparison of continuous variables between the groups according to the distribution. Categorical data were exhibited as counts and percentages and further analyzed by the Chi-squared test or Fisher’s exact test when appropriate.

Logistic regression analyses were applied to determine the independent risk factors for myocardial injury. Variables with *p* < 0.1 in the univariable analysis or those that were considered clinically relevant were entered into the multivariable models. Linear regression was applied for the assessment of the associations between cardiac biomarkers and potential risk factors. The standardized regression coefficient (R) was used to describe the association. Forest plots were applied to display the results of the multiple logistics regression analysis. A two-tailed *P* < 0.05 was considered statistically significant.

Statistical analyses were performed with SPSS 26.0 software (IBM Corp., Armonk, NY, United States). Data visualization was generated by Prism 7.0 (GraphPad Software Inc., San Diego, CA, United States).

## Results

### Baseline Characteristics in the Male and Female Patients With and Without Myocardial Injury

**FIGURE 1:**
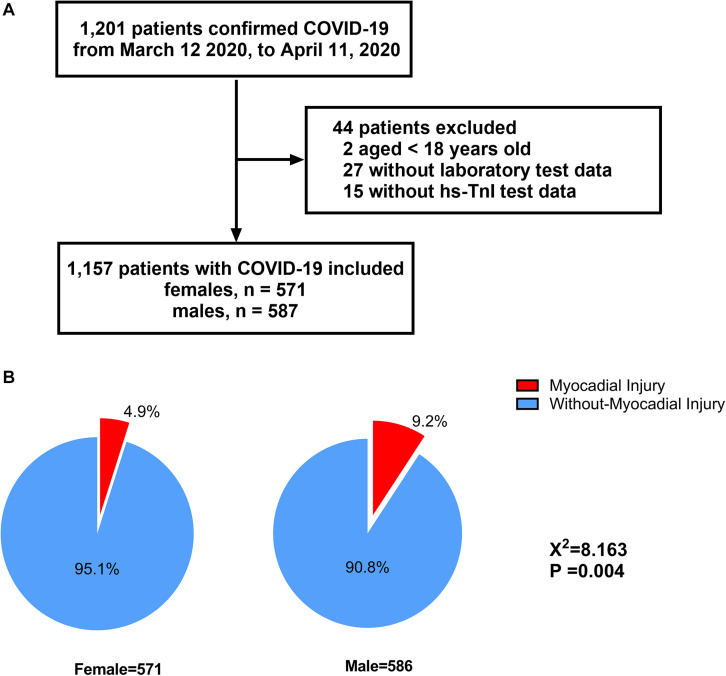
Flowchart of patients recruitment **(A)** and distribution of myocardial injury with regard to different sex inpatients with COVID-19 **(B)**.

A flow chart of the patient recruitment process is presented in Figure 1A. Briefly, a total of 1,201 COVID-19 patients were admitted to Huoshenshan Hospital from 12 March 2020 to 11 April 2020. After the exclusion of two patients aged under 18 years, 27 without laboratory test data and 15 without hs-cTnI test data, 1,157 patients were included in our final analysis, comprising 571 (49.4%) women and 586 (50.6%) men. Among the 1,157 COVID-19 patients included in our study, a significantly higher incidence of myocardial injury was observed in men than women (9.2 vs. 4.9%, *p* = 0.004) (Figure 1B).

The baseline characteristics of the patients with and without myocardial injury are summarized in Table 1. In both sexes, the presence of myocardial injury was associated with older age [women: 67.5 (57.5–75.75) vs. 61 (53–68) years, *p* = 0.004, men: 72 (63.75–78) vs. 61 (51–69) years, *p* < 0.001] and higher frequencies of hypertension [women: 15 (53.6%) vs. 193 (35.3%), *p* = 0.053; men: 35 (64.8%) vs. 177 (33.3%), *p* < 0.001] and chronic kidney disease (CKD) [women: 3 (10.7%) vs. 9 (1.7%), *p* = 0.017; men: 5 (9.3%) vs. 9 (1.7%), *p* = 0.003)].

However, in the men alone, the frequencies of coronary artery disease (CAD) [14 (25.9%) vs. 34 (6.4%), *p* < 0.001] and cerebrovascular disease [8 (14.8%) vs. 24 (4.5%), *p* = 0.004] were higher in those with myocardial injury than in those without it. Moreover, compared to their counterparts without this injury, the male myocardial injury patients had a higher incidence of nausea/vomiting [4 (7.4%) vs. 10 (1.9%), *p* = 0.039] as an initial symptom but a lower incidence of fever [29 (53.7%) vs. 391 (73.5%), *p* = 0.002]. Both in females and males, patients with myocardial injury exhibited more severe and critically ill cases and had poor clinical outcomes, such as respiratory failure, ARDS, ICU admission, and death (all *p* < 0.001).

### Laboratory Findings at Admission in Male and Female Patients With and Without Myocardial Injury

Both the male and female myocardial injury patients had higher levels of creatine kinase-MB, lactic dehydrogenase, α-hydroxybutyrate dehydrogenase, hs-cTnI, brain natriuretic peptide (BNP), myoglobin, leucocytes, high-sensitive C-reactive protein (hs-CRP), urea nitrogen, aspartate aminotransferase, prothrombin time and D-dimer but a lower lymphocyte percentage and monocyte percentage (all *p* < 0.05) (Supplementary Table S1).

### Risk Factors for Myocardial Injury in COVID-19 Patients According to Sex

**FIGURE 2:**
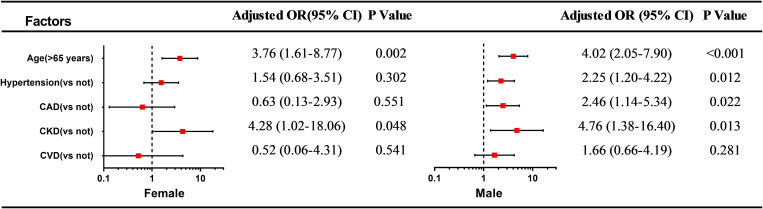
Forest plot of multivariate logistic regression analysis of age and comorbidities associated with myocardial injury in female and male COVID-19 inpatients. OR: odds ratio, 95%CI: 95% confidence intervals.

In the univariable regression analysis, age (>65 years), history of hypertension, and CKD cerebrovascular disease were risk factors for the incidence of myocardial injury in both sexes. However, in the men alone, CAD and cerebrovascular disease were associated with the incidence of myocardial injury. In the multivariable logistic regression analysis conducted among the female patients, age (>65 years) [odds ratio (OR) = 3.76, 95% confidence interval (CI) 1.61–8.77, *p* = 0.002], history of CKD (*OR* = 4.28, 95% CI 1.02–18.06, *p* = 0.048 were independent risk factors for the incidence of myocardial injury. Among the male patients, age (>65 years) (*OR* = 4.02, 95% CI 2.05–7.90, *p* < 0.001), history of hypertension (*OR* = 2.25 95% CI (1.20–4.22, *p* = 0.012), CAD (*OR* = 2.46, 95% CI 1.14–5.34, *p* = 0.022) and CKD (*OR* = 4.76, 95% CI 1.38–16.40, *p* = 0.013) were independently associated with the incidence of myocardial injury (Supplementary Table S2 and Figure 2). In terms of laboratory variables, after multivariable adjustment for age and the above-mentioned comorbidities, the leucocyte count (*OR* = 3.13, 95% CI 1.24–7.86, *p* = 0.016), the levels of hs-CRP (*OR* = 4.45, 95% CI 1.33–14.83, *p* < 0.001) and D-dimer (*OR* = 3.93, 95% CI 1.27–12.19, *p* = 0.018) were determined as being independently related to myocardial injury only in the male patients (Table 2).

### Correlations Between hs-CRP, D-Dimer and Biomarkers of Myocardial Injury in COVID-19 Patients According to Sex

**FIGURE 3:**
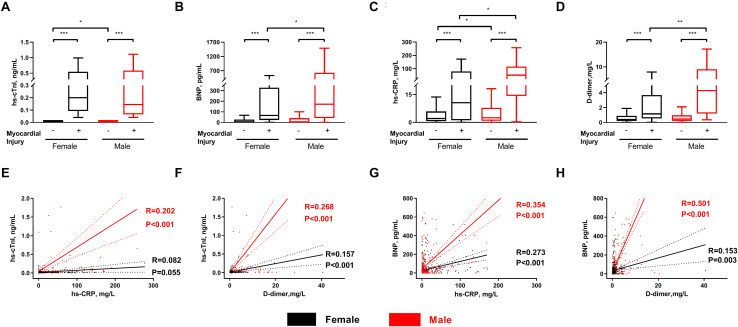
Comparison and correlation of the laboratory testings in COVID-19 inpatients with and without myocardial injury. Comparison of the level of **(A)** hs-cTnI, **(B)** BNP, **(C)** hs-CRP, and **(D)** D-dimer between females and males with and without myocardial injury; Correlation between hs-CRP with hs-TnI **(E)**, D-dimer with hs-TnI **(F)**, hs-CRP with BNP **(G)**, and D-dimer with BNP **(H)** in females and males. Hs-cTnI: high-sensitive cardiac troponin I; BNP: brain natriuretic peptide, hs-CRP: high-sensitive C-reactive protein. *p < 0.05, **p < 0.01, ***p < 0.005.

Compared to the female myocardial injury patients, the male patients had a remarkable increase in levels of BNP [167.71 (38.47–611.47) vs. 59.13 (6.25–305.97) pg/mL, *p* < 0.05], hs-CRP [51.07 (14.27–115.39) vs. 10.57 (1.13–79.22) mg/L, *p* < 0.05] and D-dimer [4.29 (1.20–9.05) vs. 1.16 (0.54–3.69) mg/L, *p* < 0.01]. However, hs-cTnI level did not manifest the significant sex difference in myocardial injury patients (*p* > 0.05) (Figure 3).

In the linear regression analysis, the levels of hs-CRP were positively correlated with those of hs-cTnI in the men (*R* = 0.202, *p* < 0.001) but not women. Furthermore, the levels of D-dimer were correlated with the hs-cTnI values in both the men (*R* = 0.268, *p* < 0.001) and women (*R* = 0.157, *p* < 0.001). The levels of hs-CRP (men: *R* = 0.354, *p* < 0.001; women: *R* = 0.273, *p* < 0.001) and D-dimer (men: *R* = 0.501, *p* < 0.001; women: *R* = 0.153, *p* = 0.003) were correlated with those of BNP in both sexes. However, the correlations were stronger in the men than women (Figure 3).

## Discussion

In the present study, we found that the incidence of myocardial injury was higher among male COVID-19 patients than their female counterparts. The multivariate logistic regression analyses showed that old age and CKD were independently associated with the presence of myocardial injury in both sexes. However, history of hypertension and CAD, the elevated hs-CRP and D-dimer levels were independent risk factors only in the men. Additionally, while correlations of hs-CRP and D-dimer with cardiac biomarkers were observed in both sexes, they were more pronounced in men. Our findings suggest the presence of sex differences in the incidence and risk factors of myocardial injury in COVID-19 patients.

### Sex Differences in the Incidence of Myocardial Injury and Impacts on Cardiac Function

As illustrated above, the sex bias in the incidence of myocardial injury in COVID-19 patients is controversial. Although some studies reported the absence of significant sex differences in the incidence of myocardial injury (Lombardi et al., 2020; Shi et al., 2020b), our large-scale study suggested that men are likelier to develop it. Interestingly, the absolute value of hs-TnI did not manifest the sex disparities in myocardial injury patients, suggested that no sex difference existed in the severity of myocardial injury even if males were more prone to it. Similarly, a study of 2,736 COVID-19-positive individuals in New York City reported that no significant sex differences when the severity of myocardial injury was stratified by troponin I degrees (Lala et al., 2020).

In the present study, those with myocardial injury showed a marked increase in their BNP levels in association with serious cardiac function impairment (Troughton et al., 2014). Previous studies that focused on the cardiovascular implications of COVID-19 found that the mean concentration of BNP was much higher in those who died, highlighting the prognostic significance of this parameter (Guo et al., 2020). Accordingly, it can be hypothesized that patients with a higher risk of severe COVID-19 progression and outcome due to myocardial injury and worse cardiac function may include a disproportionate number of males.

### Sex Differences in Risk Factors for Myocardial Injury

Consistent with previous studies (McCarthy et al., 2019; Shi et al., 2020a), the myocardial injury patients in our study tended to be older and have a larger number of pre-existing illnesses (hypertension and CKD) suggesting that these comorbidities accelerate the development of myocardial injury. Furthermore, male patients were older and had higher incidences of CAD and cerebrovascular disease. In line with our findings, Guo et al. reported that the proportions of men were higher than those of women in elderly populations and populations with coronary heart disease (Guo et al., 2020).

Generally, elderly males suffer from a more serious reduced in total numbers of immune cells and inverted CD4/CD8 T-cell ratio as compared with the female (Perrotta et al., 2020), resulting in the impaired immunologic surveillance and immune clearance function in aging males. Moreover, Svartengren et al. (2005) demonstrated the clearance function of inhaled particles in small airway areas decreased with age. In addition, upper airway size as well decreases in an age-related manner, which is more pronounced in males (Martin et al., 1997).

These viewpoints partly account for the sex differences in COVID-19 infection rate and are indispensable to the further interpretation of the higher myocardial incidence in aging males compared to aging females (Perrotta et al., 2020).

### Sex Differences in the Mechanism of Myocardial Injury

To characterize cardiac structural and functional abnormalities of COVID-19 patients, echocardiographic and electrocardiographic data have been analyzed by several researchers. Giustino et al. (2020) reported that patients with myocardial injury more suffered from left ventricle dysfunction, regional wall motion abnormalities, right ventricle dysfunction, and pericardial effusions. Additionally, recently researches assessed by speckle-tracking echocardiography supported that worsening left ventricle and right ventricle function, reflected by reduced global and regional strain, were more observed in patients with severe COVID-19 infection and more associated with poorer grade and clinical deterioration (Lassen et al., 2020; Rothschild et al., 2020). Meanwhile, 12-lead electrocardiogram identified that two different patterns of ST-segment changes, including global biventricular dysfunction related diffuse ST-segment changes and regional wall motion abnormalities associated regional ST-segment changes (Giustino et al., 2020). It was worth noting that the sex differences in cardiac structural and functional characteristic changes were not been reported.

To an extent, the above findings partly accounted for the potential pathophysiological mechanism of myocardial injury caused by COVID-19, such as direct viral invasion and possibly ischemia-reperfusion injury of the myocardium. Nevertheless, a much larger body of literature suggests that the high degree of systemic inflammation and microvascular thrombosis mediated by the cytokine release syndrome in hospitalized COVID-19 patients may be more principal in the development of myocardial injury (Akhmerov and Marbán, 2020; Colling and Kanthi, 2020). In SARS-CoV-2 infection, the abnormal release of proinflammatory factors could cause endothelial cell apoptosis, resulting in immunopathogenic damage to the cardiovascular system (Teuwen et al., 2020). These factors may shift the balance of coagulation toward a procoagulant and prothrombotic state (Corrales-Medina et al., 2013). Consistently, our study demonstrated that both the male and female patients with myocardial injury presented abnormal inflammation and coagulation stress, as suggested by the higher levels of hs-CRP and D-dimer, and developed elevated leukocyte counts and neutrophil percentages. We also observed that the hemoglobin level was decreased in those with myocardial injury. Taking into account the oxygen-carrying capacity of hemoglobin and cardiac oxygen metabolism imbalance, the latter may be of particular significance in the development of myocardial injury and early prediction of disease prognosis.

Furthermore, our findings add value to those of previous studies by demonstrating that the levels of hs-CRP and D-dimer in men with myocardial injury were almost five and threefold higher than those in the women, respectively. The sexual dimorphism in the hyperinflammatory state may be mediated by different innate and adaptive immune responses based on sex chromosomes (Klein and Flanagan, 2016; Schurz et al., 2019). A large number of immune-related genes located in the X chromosome confer upon women a stronger degree of immune recognition and a higher elimination rate of pathogenic agents (Schurz et al., 2019). As observed in a study that enrolled 331 COVID-19 patients, critically ill female patients have significantly higher levels of SARS-CoV-2 IgG antibodies than their male counterparts (Zeng et al., 2020).

### Sex Differences in the Association Between hs-CRP, D-Dimer, and Cardiac Biomarkers

Interestingly, our study supports the notion of the presence of an independent risk relationship of the inflammatory response and coagulation disorder with myocardial injury and cardiac dysfunction in male rather than female patients. It indicated that the men experienced more severe COVID-19 infection, and were more susceptible to inflammation and coagulation stress. Angiotensin-converting enzyme 2 (ACE2) mediates the entry of the virus into host cells by binding with the virus spike protein. However, this process results in the downregulation of ACE2 as well as uncontrolled renin-angiotensin-aldosterone system activation and further myocardial adverse outcomes (Nishiga et al., 2020). Of note, the ACE2 gene, located on the X chromosome, might experience differences in methylation with sex-chromosome activation (Ambrosino et al., 2020), which probably increased the possibility of sex-oriented susceptibility of myocardial injury.

Meanwhile, women have a higher level of estrogen, which enhances the level of ACE2 activity and expression in a concentration-dependent manner (Rice et al., 2004), upregulates the expression of angiotensin-(1–7) and prompts vasodilation, NO release and reduced smooth muscle cell proliferation (Ji et al., 2008). Estrogen as well exhibits a protective effect against the vascular endothelial injury caused by inflammation (Chakrabarti et al., 2014). Under oxidative stress, estrogen reduces the rate of reactive oxygen species generation by specific posttranslational modifications in the mitochondrial enzymes, inducing a lower rate of myocardial injury in women (Lagranha et al., 2010). Accordingly, we hypothesized that the inflammation reaction and coagulation state vary according to sex and female-specific protective mechanisms, probably mediating sex differences in the incidence of myocardial injury and resulting in sex differences in the incidence of adverse outcomes in COVID-19 patients.

### Limitations

Our study has some limitations. Firstly, data on virus antibodies and proinflammatory cytokines (e.g., interleukin [IL]-1, IL-6, IL-8 and tumor necrosis factor-α) were not available, which would provide a proper insight into the pathophysiological stage of the myocardial injury from viral infection to the immune reaction. Moreover, the widespread application of echocardiography was limited due to the rapid progress of the emergency in Wuhan and the consideration of biosafety protection measures for hospital staff. Echocardiographic data were available only in partial subjects and were not analyzed in our retrospective research. Next, this study had a single-center design; our findings require validation in further rigorous prospective studies. Fourthly, our study was retrospective in nature and could only speculate the biological relationship between sex differences and myocardial injury on the basis of our evidence and that of previous studies.

## Conclusion

Our results suggest that the incidence of myocardial injury in COVID-19 patients is sex-dependent, predominantly in association with a higher degree of inflammation and coagulation disorder in men. These findings may provide a reasonable explanation for the observed sex differences in the adverse outcomes in COVID-19 patients and a theoretical basis for sex-based clinical trials and management.

## Data Availability Statement

All datasets generated for this study are included in the article/Supplementary Material, further inquiries can be directed to the corresponding author/s.

## Ethics Statement

The studies involving human participants were reviewed and approved by the Human Ethics Committee, Huoshenshan Hospital (No. HSSLL023). Written informed consent for participation was not required for this study in accordance with the national legislation and the institutional requirements.

## Author Contributions

RC, CL, and JY contributed to the study design and drafting of the manuscript. LH revised the final manuscript. HT, XD, LZ, and PL contributed to the data collection. RC, CL, JY, YY, YS, RZC, XG, JK, FY, and CH analyzed the data. All authors read and approved the final manuscript.

## Conflict of Interest

The authors declare that the research was conducted in the absence of any commercial or financial relationships that could be construed as a potential conflict of interest.
