# Supplementary material for: Sex Differences in the Incidence and Risk Factors of Myocardial Injury in COVID-19 Patients: A Retrospective Cohort Study
Source: Front Physiol. 2021 Feb 16;12:632123. doi: 10.3389/fphys.2021.632123 (PMC7920972; doi:10.3389/fphys.2021.632123)
Supplement: Supplementary file 1 [file Data_Sheet_1.docx]

Supplementary Material

**Supplemental Table S1: Comparison of laboratory findings between myocardial injury and without-myocardial injury in female and male COVID-19 patients**

|  |  | **Female** | | **p value** | **Male** | | **p value** |  |
| --- | --- | --- | --- | --- | --- | --- | --- | --- |
|  | **Nomal Range** | **Myocardial Injury (n=28)** | **Without-Myocardial Injury (n=543)** |  | **Myocardial Injury (n=54)** | **Without-Myocardial Injury (n=532)** |  |  |
| **Cardiac biomarkers** |  |  |  |  |  |  |  | |
| Creatine kinase, IU/L | 50-310 | 45.20 (33.20-157.00) | 42.90 (31.88-61.66) | 0.083 | 92.85 (46.85-180.48) | 51.65 (35.19-72.33)^##^ | <0.001 | |
| Creatine kinase-MB, IU/L | 0-24 | 12.30 (10.00-19.00) | 8.45 (6.90-10.90) | <0.001 | 13.33 (9.83-19.83) | 8.70 (6.98-11.05) | <0.001 | |
| Lactic dehydrogenase, IU/L | 120-250 | 326.30 (198.10-402.21) | 179.83 (153.18-215.85) | <0.001 | 290.15 (229.35-398.05) | 184.00 (156.51-217.75) | <0.001 | |
| α-hydroxybutyrate dehydrogenase, IU/L | 72-182 | 264.05 (163.10-368.86) | 147.15 (124.10-176.44) | <0.001 | 235.74 (179.65-320.33) | 148.2 (127.13-177.68) | <0.001 | |
| hs-cTnI, ng/mL | 0-0.04 | 0.20 (0.09-0.55) | 0.01 (0.01-0.01) | <0.001 | 0.15 (0.07-0.58) | 0.01 (0.01-0.01)^##^ | <0.001 | |
| BNP, pg/ml | 0-100 | 59.13(6.25-305.97) | 0.01 (0.01-27.47) | <0.001 | 167.71 (38.47-611.47)^*^ | 0.01 (0.01-40.45) | <0.001 | |
| Myoglobin,ng/mL | 0-65 | 7.32 (0.18-21.54) | 4.13 (1.98-6.82) | 0.035 | 47.71 (9.24-210.68)^*^ | 6.50 (3.48-10.53)^##^ | <0.001 | |
| **Blood routine test** |  |  |  |  |  |  |  | |
| Leucocytes, ×10^9^/L | 4-10 | 6.80 (4.86-10.38) | 5.50 (4.60-6.90) | 0.048 | 6.80 (5.43-11.28) | 5.80 (4.80-7.18) | <0.001 | |
| Neutrophil percentage, % | 40-75 | 69.05 (60.1-86.88) | 61.20 (54.60-66.80) | 0.002 | 84.11 (68.33-89.14)^*^ | 63.50 (56.10-70.55)^##^ | <0.001 | |
| Lymphocyte percentage, % | 20-50 | 15.75 (6.96-30.25) | 28.20 (23.00-34.20) | <0.001 | 9.35 (5.78-21.08) | 24.90 (19.10-31.50)^##^ | <0.001 | |
| Eosnophil percentage, % | 0.4-8 | 0.75 (0.10-2.17) | 2.00 (1.20-3.20) | <0.001 | 1.23 (0.20-2.16) | 2.10 (1.10-3.40) | <0.001 | |
| Basophil percentage, % | 0-1 | 0.19 (0.10-0.48) | 0.40 (0.30-0.55) | <0.001 | 0.20 (0.05-0.40) | 0.40 (0.20-0.50) | <0.001 | |
| Monocyte percentage, % | 3-10 | 6.65 (4.73-7.62) | 7.40 (6.30-8.70) | 0.017 | 5.95 (3.53-7.61) | 7.83 (6.30-9.30)# | <0.001 | |
| Hemoglobin, g/L | 115-150 | 112 (100-129) | 118 (109-128) | 0.197 | 117 (100-127) | 127 (118-140)^##^ | <0.001 | |
| Platelets, ×10^9^/L | 100-300 | 208(152-271) | 230 (193-279) | 0.100 | 188 (108-262) | 221 (183-269) | 0.001 | |
| **Inflammatory biomarker** |  |  |  |  |  |  |  | |
| hs-CRP, mg/L | 0-4 | 10.57 (1.13-79.22) | 2.12 (0.74-5.92) | <0.001 | 51.07 (14.27-115.39)^*^ | 2.49 (0.89-7.89)^#^ | <0.001 | |
| **Liver function** |  |  |  |  |  |  |  | |
| ALT, IU/L | 7-50 | 18.95 (14.95-30.74) | 19.00 (12.70-30.96) | 0.570 | 27.76 (16.63-49.50) | 27.20 (17.90-42.80)^##^ | 0.960 | |
| AST, IU/L | 4-45 | 21.30 (17.73-31.78) | 18.80 (15.20-25.10) | 0.027 | 30.10 (20.73-43.06)^*^ | 21.10 (16.00-28.90)^##^ | <0.001 | |
| TBil, μmol/L | 0-26 | 10.40 (7.40-13.70) | 8.60 (6.70-11.20) | 0.017 | 11.45 (8.48-15.06) | 9.90 (7.80-12.83)^##^ | 0.019 | |
| **Renal function** |  |  |  |  |  |  |  | |
| Urea nitrogen, mmol/L | 3.1-9.5 | 5.11 (3.88-8.48) | 4.20 (3.45-5.13) | 0.002 | 7.15 (5.32-12.79)^*^ | 4.66 (3.78-5.72)^##^ | <0.001 | |
| Creatinine, μmol/L | 41-100 | 65.55 (52.68-89.75) | 57.20 (51.25-65.70) | 0.014 | 78.40 (64.43-122.97)^*^ | 70.80 (61.95-79.97)^##^ | 0.002 | |
| **Coagulation profiles** |  |  |  |  |  |  |  | |
| PT, s | 9.2-18 | 13.55 (12.16-15.39) | 12.62 (12.02-13.38) | 0.008 | 14.10 (13.31-16.46) | 13.03 (12.42-13.84)^##^ | <0.001 | |
| APTT, s | 21-40 | 28.55(26.31-30.47) | 27.41(25.71-29.57) | 0.240 | 28.63(26.74-32.59) | 28.355(26.27-30.60)^##^ | 0.265 | |
| D-dimer, mg/L | 0-0.6 | 1.16 (0.54-3.69) | 0.41 (0.20-0.89) | <0.001 | 4.29 (1.20-9.05)^**^ | 0.46 (0.21-0.97) | <0.001 | |

Continuous variables with non-normal distribution were represented as median (25th-75th quartile) or mean ± SD. Hs-CRP: high-sensitive C-reactive protein, ALT: alanine aminotransferase, AST: aspartate aminotransferase, TBil: total bilirubin, hs-cTnI: high-sensitive cardiac troponin I, BNP: brain natriuretic peptide, PT: prothrombin time, APTT: activated partial thromboplastin time. *p<0.05, **p<0.01 compare to female patients with myocardial injury. #p<0.05, ##p<0.01 compare to female patients without myocardial injury.

**Supplemental Table S2: Univariate and Multivariate logistic regression analysis of age and comorbidities associated with myocardial injury in female and male COVID-19 patients.**

| Variables | **Female** | | | |  | **Male** | | | |
| --- | --- | --- | --- | --- | --- | --- | --- | --- | --- |
|  | **Univariable**  **OR (95% CI)** | **p value** | **Multivariable**  **OR (95% CI)** | **p value** |  | **Univariable**  **OR (95% CI)** | **p value** | **Multivariable**  **OR (95% CI)** | **p value** |
|  |  |  |  |  |  |  |  |  |  |
| Age (>65 years vs ≤65 years) | 4.26 (1.89-9.60) | <0.001 | 3.76 (1.61-8.77) | 0.002 |  | 5.78 (3.06-10.9) | <0.001 | 4.02 (2.05-7.90) | <0.001 |
| Comorbidities( vs not present ) | |  |  |  |  |  |  |  |  |
| Hypertension | 2.09 (0.98-4.49) | 0.058 | 1.54 (0.68-3.51) | 0.302 |  | 3.70 (2.05-6.64) | <0.001 | 2.25 (1.20-4.22) | 0.012 |
| Diabetes | 1.90 (0.78-4.62) | 0.156 | Not selected | |  | 1.54 (0.79-2.98) | 0.204 | Not selected | |
| Arrhythmia | 0.80 (0.10-6.14) | 0.831 | Not selected | |  | 2.05 (0.67-6.23) | 0.206 | Not selected | |
| Malignant neoplasm | 1.64 (0.21-13.07) | 0.641 | Not selected | |  | 2.18 (0.61-7.83) | 0.234 | Not selected | |
| CAD | 1.05 (0.24-4.61) | 0.946 | 0.63 (0.13-2.93) | 0.551 |  | 5.13 (2.54-10.33) | <0.001 | 2.46 (1.14-5.34) | 0.022 |
| COPD | 1.30 (0.17-10.18) | 0.805 | Not selected | |  | 1.41 (0.48-4.17) | 0.537 | Not selected | |
| CLD | 2.20 (0.27-17.98) | 0.463 | Not selected | |  | 1.43 (0.41-4.96) | 0.572 | Not selected | |
| CKD | 7.12 (1.82-27.93) | 0.005 | 4.28 (1.02-18.06) | 0.048 |  | 5.93 (1.91-18.39) | 0.002 | 4.76 (1.38-16.40) | 0.013 |
| Anemia | 1.97 (0.24-15.99) | 0.524 | Not selected | |  | 3.85 (0.99-14.98) | 0.052 | Not selected | |
| cerebrovascular disease | 1.08 (0.14-8.40) | 0.941 | 0.52 (0.06-4.31) | 0.541 |  | 3.68 (1.57-8.66) | 0.003 | 1.66 (0.66-4.19) | 0.281 |

OR: odds ratio, 95% CI: 95% confidence intervals. CAD: coronary artery disease, COPD: chronic obstructive pulmonary disease, CLD: chronic liver disease, CKD: chronic kidney disease
